# Complementing two-photon fluorescence detection with backscatter detection to decipher multiparticle dynamics inside a nonlinear laser trap

**DOI:** 10.1038/s41598-022-27319-z

**Published:** 2023-01-13

**Authors:** Anita Devi, Sumit Yadav, Arijit K. De

**Affiliations:** 1 Present Address: Condensed Phase Dynamics Group, Department of Physical Sciences, Indian Institute of Science Education and Research (IISER) Mohali, Knowledge City, Sector 81, SAS Nagar, Manauli, Punjab 140306 India; 2 Condensed Phase Dynamics Group, Department of Chemical Sciences, Indian Institute of Science Education and Research (IISER) Mohali, Knowledge City, Sector 81, SAS Nagar, Manauli, Punjab 140306 India; 3 Present Address: Department of Physics, University of Alberta, Edmonton, AB T6G 2R3 Canada

**Keywords:** Nonlinear optics, Optical manipulation and tweezers

## Abstract

Using wide-field and point detection modalities, we show how optical trapping dynamics under femtosecond pulsed excitation can be explored by complementing detection of two-photon fluorescence with backscatter. Radial trajectories of trapped particles are mapped from correlated/anti-correlated fluctuations in backscatter pattern whereas temporal evolution of two-photon fluorescence is used to mark the onset of trapping involving multiple particles. Simultaneous confocal detection of backscatter and two-photon fluorescence estimates axial trap stiffness, delineating short-time trapping dynamics. When a second particle is being trapped an oscillatory signal is observed which is due to interference of backscatter amplitudes, revealing inter-particle interactions within the trap. These findings are crucial steps forward to achieve controlled manipulation by harnessing optical nonlinearity under femtosecond pulsed excitation.

The advent of mode-locked oscillators, producing picosecond and femtosecond pulses at high repetition-rates (> 1 MHz)^1^, enabled facile observation of nonlinear optical phenomena at much low average power (compared with continuous-wave or CW lasers) having wide-ranging applications in microscopy and imaging^2^. Although in the context of optical trapping and manipulation using laser tweezers^3,4^ advantages of femtosecond pulsed excitation harbored on detection of fluorescence due to two-photon absorption (TPA)^5^ (i.e. two-photon fluorescence, TPF) was demonstrated^6–10^, much in the same way as in TPF microscopy^11^, it was only recently that the concept of nonlinear optical force on dielectric particles, arising from third-order optical susceptibility (i.e. from optical Kerr effect, OKE), was put forward^12–14^; it was shown that under ultrashort pulsed excitation a delicate balance between gradient and scattering forces, fine-tuned by OKE, leads to existence of an optimal laser power corresponding to the most stable optical trap, refuting the widely accepted rule-of-thumb that higher laser power is required for better trapping efficiency. Parallel to these theoretical endeavors, using multimodal laser tweezers^15^ such modulation of trapping potential under pulsed excitation was experimentally mapped from bright-field image and backscatter signal analysis, corroborated by numerical results incorporating optical as well as thermal nonlinearities^16^; one crucial finding was that a steep rise in signal followed by a decrease as the particle is first dragged to the potential energy minimum under the action of nonlinear optical force and subsequently shelved into a new minimum due to retarded action of thermal nonlinearity (‘adjustment dynamics’, explained later) and the trapped particle’s dynamics is further modified^16^. As already mentioned, TPF detection was utilized to probe optical trapping of fluorescent nanoparticles or fluorophore-coated microparticles^7–10,16^; however enhanced photobleaching (i.e. light-induced transformation of the fluorophore into a non-fluorescent photo-product) associated with TPA^17^ needs some more attention as it would otherwise lead to erroneous interpretation of data. In fact, TPF detection of optical trapping of 100 nm polystyrene particles under femtosecond pulsed excitation showed rapid decay of TPF signal over time (due to photo-bleaching) although it is much more sensitive in detecting single nano-particle/cluster in a background-free manner^18^. This has motivated us to record and analyze backscatter signal and TPF signal in parallel as they complement each other which we present in this article. Using polystyrene microspheres under femtosecond pulsed excitation, we first investigate optical trapping dynamics by analyzing TPF and backscatter images with high spatial resolution and discuss the merits and limitations associated with detection of either type of signal. We further demonstrate how simultaneous detection of TPF and backscatter with high temporal resolution can capture nonlinear trapping dynamics in a much more comprehensive way.

In the experiment, we use fluorophore-coated polystyrene beads of 1 µm diameter (F8819, Thermo Fisher Scientific) suspended in water. A 800 nm pulsed laser beam from a Ti–Sapphire oscillator (Vitesse, 2 W, Coherent Inc.) with a repetition rate of 80 MHz is used for optical trapping using a home-built optical tweezers set-up^15^. A schematic of the experimental set-up is shown in Section S1 in Supplementary Information (SI). It is equipped with flexible detection modalities: wide-field detection mode to capture images using CMOS camera (DCC1645C, Thorlabs Inc.) and point detection mode to record signal using photomultiplier tubes (PMTs; PMM01 and PMM02, Thorlabs Inc.). In wide-field detection mode we record either TPF image or backscatter image (dark-field mode) or transmitted white light images (bright-field mode), not presented in this article. In point detection mode, we simultaneously detect both TPF signal and backscatter signal. The TPF image/signal are collected by using a 680 nm short-pass filter (FF01-680/SP-25, Semrock Inc.) and backscatter image/signal is collected using a 776 nm long-pass filter (FF01-776/LP-25, Semrock Inc.). The dark-field images are captured at 25 frames per second (fps) and PMT data are collected at 400 µs as well as at 400 ns intervals. The quantum efficiency of PMT detecting TPF is around 2% at 575 nm wavelength, and the exposure time of the CMOS camera is around 39.9 ms. The pulse width at sample position is ~ 500 fs (measured by collinear TPF autocorrelation^15^) which varied little from day-to-day measurements. All experiments were performed at room temperature (25 °C); data recording/analysis was done using laboratory-built programs coded with LabVIEW (National Instruments Inc)/Matlab (Mathworks Inc) programming and open-source ‘Physics Tracker’ software was used for analyzing CMOS camera images.

First, we present analysis of TPF and backscatter CMOS camera images for single-particle and multiparticle dynamics.

A detailed discussion on radial intensity profiles of TPF image of a single trapped particle is provided in Section S2.1 in SI and the Video 1 captures a live event of single-particle trapping. As shown in Fig. 1a, when a particle is dragged to the trap center, there is an immediate rise in the TPF signal followed by a decay due to photobleaching. To ensure that the observed decay is due to photobleaching, we measure the time-dependent TPF intensity from a immobilized particle (stuck on the coverslip) as well for quantitative comparison of photobleaching timescales; we notice melting/ablation following an initial decay in TPF at high power indicated by sudden drop in signal, as evident from Fig. 1b and Video 2. The absence of any melting/ablation during trapping is due to dissipation of heat (caused by laser-induced heating of particle) by surrounding medium (water). With increasing average power, photobleaching rate becomes faster; a detailed discussion is given in Section S2.2.1 in SI. Figure 1c shows the TPF signal when two particles are trapped one after another. Due to photobleaching of the first particle, from such a TPF trace it cannot be ascertained whether the first particle is still trapped while the second one is being trapped. However, the sudden rise in TPF signal turns out to be advantageous to mark the onset of trapping during multiparticle trapping, as shown in Fig. 1d.

**Figure 1:**
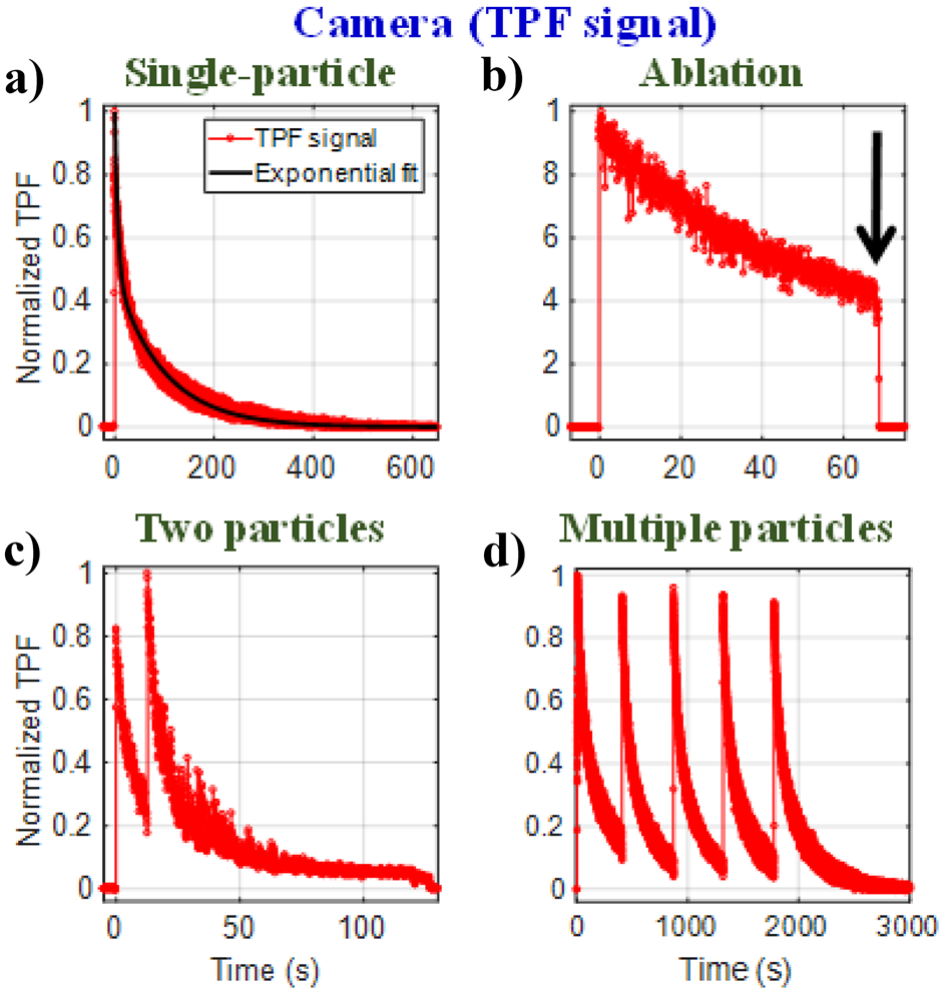
TPF signal from (**a**) trapped single-particle, (**b**) ablation for stuck particle (indicated by an arrow), (**c**) two-particles and (**d**) multiple particles.

A ‘four-lobed’ image, which reflects typical quadrupolar scatter pattern when the particle-size is comparable with the wavelength of trapping beam^15,19^, from a single trapped particle is shown in Fig. S5a (in Section S2.3.1.1 in SI). By analyzing the intensity for each lobe, we can determine the radial direction of motion (with respect to polarization of electric field of laser) of the trapped particle at any instant of time. As discussed in detail in a previous work^16^, the analysis of overall backscatter intensity (summed over all lobes) shows how the confinement time (time during which the particle stays inside the trap) changes with average laser power, thus furnishing information on the stability of the trap which is inaccessible from TPF trace due to photobleaching. A detailed discussion on single-particle dynamics based on backscatter signal analysis is given in S2.3.1.2 in SI. Figure 2a shows a time-series backscatter images when two particles are confined within the trap; a live event can be seen in Video 3. It should be noted that compared with one-particle scatter pattern (S2.3.1.1 in SI) the intensity changes are more when two particles are trapped. Since more than two particles cannot be accommodated inside the focal volume (the diameter of the focused Gaussian beam, i.e. the beam-waist, being ~ 570 nm), when one particle is confined within the trap center the other one must be positioned slightly behind/ahead along the axial direction (or both should be positioned across the trap center). Due to convergence/divergence of the laser beam, the particle(s) positioned behind/ahead of the trap center would experience a large amplitude motion along radial direction. Thus, the origin of the change in intensity of each lobe may be explained by the motion of the two particles inside the trap, causing a change in the radial projection, which is depicted in Fig. 2b. A detailed discussion on multiparticle dynamics based on backscatter signal analysis is given in Section S2.3.2.1 in SI. From the overall backscatter intensity (summed over all lobes) during trapping of multiparticles shown in Fig. S9 (in Section S2.3.2.2) in SI, it is evident that the likelihood of two particles being trapped depends on the initial momentum of the second particle or, equivalently, the average laser power. It can also be noted that when one particle replaces the other, there is a slight kink in the backscatter trace (Fig. S9a), as opposed to sharp spike in TPF trace (Fig. 1c,d).

**Figure 2:**
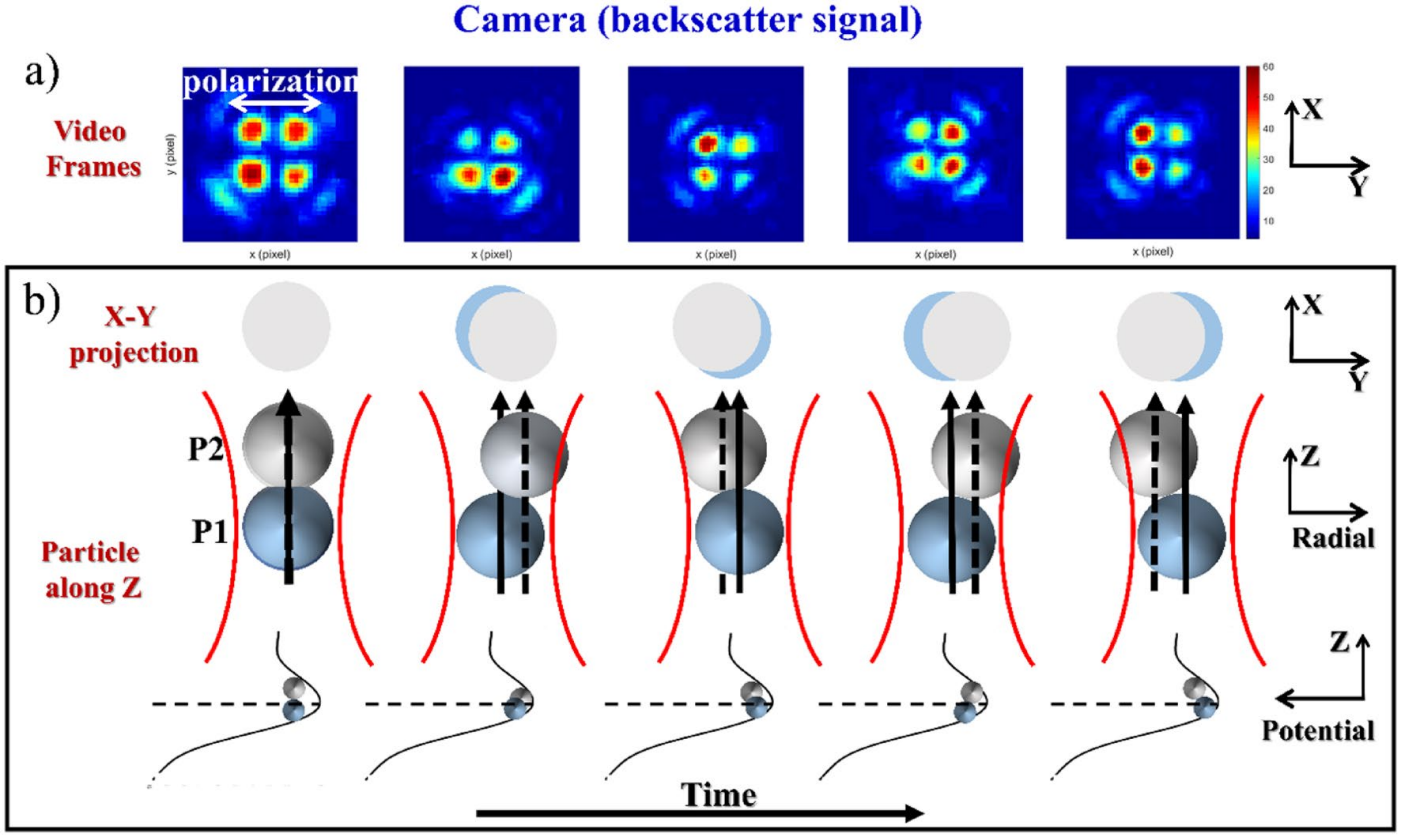
Two-particle backscatter pattern changing over time where the dashed arrow shows lateral deviation from the beam axis (shown as solid arrow); horizontal dotted line represents the focal plane (z = z_0_) under pulsed excitation. The first row is the scattering pattern captured while two particles are trapped within the focal volume (each images is of 30 pixel × 30 pixel in dimension with each pixel having an area of 3.60 µm × 3.60 µm). The second row represents a proposed model depicting the X–Y projection of trapped particles corresponding to the axial alignment of these particles inside the potential well shown in third and fourth rows.

From the preceding discussion, it is imperative that we analyze both TPF and backscatter in order to have complementary information on trapping dynamics. This led us detect both types of signals simultaneously using PMTs with much improved temporal resolution compared with that using CMOS camera; in addition, confocal detection of backscatter suppresses contributions from out-of-focus background, thereby greatly improving the signal-to-noise. While this approach is at the expense of spatial resolution, in the present study the main focus was to capture fast events in trapping dynamics (without any attempt to measure short-time trap-stiffness which would require combined spatio-temporal resolution, for example, to capture the transition from ballistic to diffusive regimes^20^).

From the time traces of backscatter, following an initial spike (caused by drag to the trap center by optical force) a fast decay is observed which corresponds to the particle’s motion along the axial direction to the shifted trap center caused by retarded (compared to optical force) action of thermal nonlinearity and was termed as ‘adjustment dynamics’^16^; note that this behavior is not observed while using the camera because of the low frame rates. To verify whether the TPF signal also captures such an event, we fit the TPF decay traces to three exponentials; the presence of intermediate emissive species during light bleaching justifies the widely used method of estimating photo bleaching kinetics by multiple exponentials. Comparing these time constants (Table S5 in Section S3.1 in SI) with those obtained from backscatter traces, it may be inferred that the time constant with few milliseconds correspond to the adjustment dynamics while the other two components (of few seconds and few tens of seconds) correspond to photobleaching dynamics. However, quantitative estimation of timescale for the adjustment dynamics from TPF data would be erroneous since it is an arduous task to disentangle these timescales. The confinement time of the particle in trap is decreases with average power and the escape potential can be mapped from this data as shown in Fig. S11, and a discussion of data analysis is provided in Section S3.1.1 in SI (also see reference 16 for a detailed discussion).

Now, in order to measure trap stiffness, we analyze both backscatter and TPF signal over the same time interval during which a single-particle is trapped. The baseline for each signal is subtracted and power spectrum is constructed from the Fourier transform of the residual^21–23^, as shown in Fig. 3 (described in detail in Section S3.1.2 in SI). To estimate the power spectrum data (PSD) from the FFT spectrum, we use the following relation:

$$PSD= \frac{{\left|FFT\left(residual\right)\right|}^{2}}{L},$$

Here, $L$ is the length of the vector. Finally, we plotted the PSD in the ${log}_{10}$ scale. The result from each step is shown in Fig. S12.

**Figure 3:**
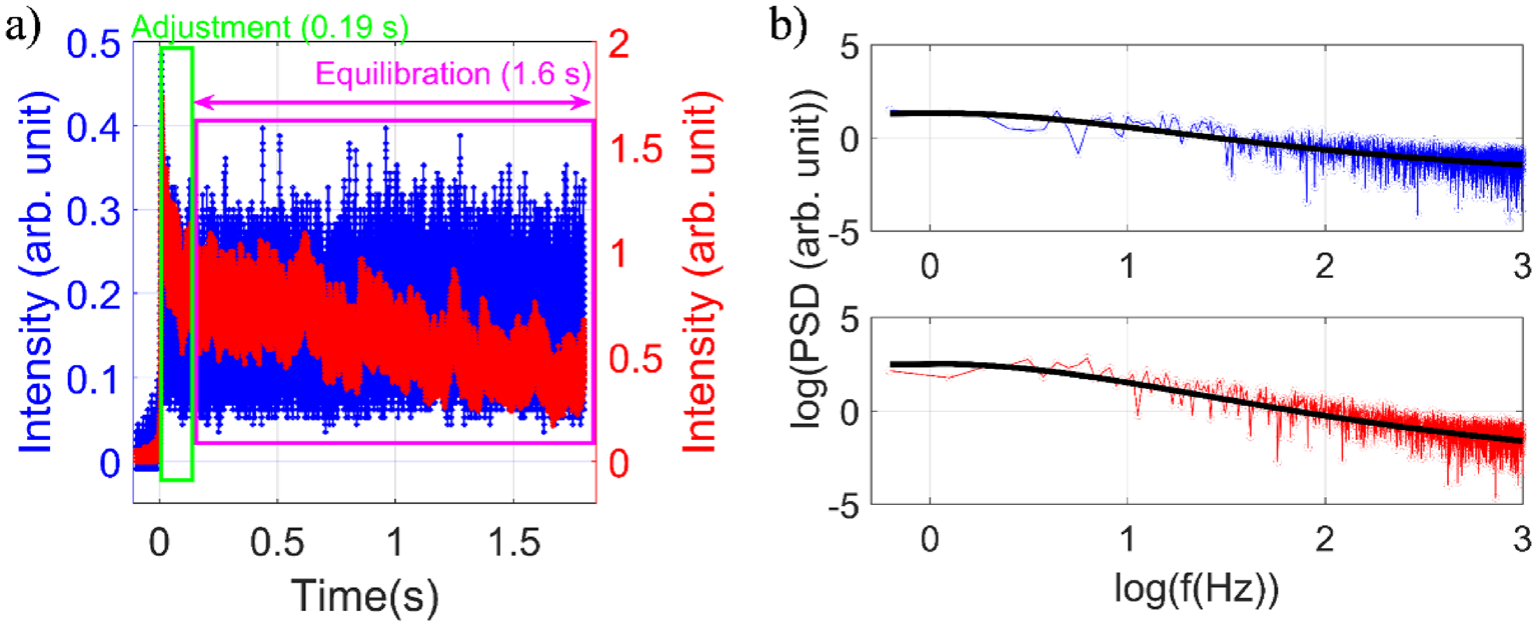
Plots of (**a**) backscatter and TPF signals for single trapped particle, and (**b**) corresponding power spectra at 18.80 mW average power under pulsed excitation. Color: red corresponds to the TPF signal, blue corresponds to the backscatter signal, and black corresponds to Lorentzian fit to the power spectrum.

For analyzing TPF signal, we consider data for a time window of ~ 2 s (following the initial adjustment time). We fit the data in two exponentials with an additional constant since we do not capture the complete photobleaching dynamics within 2 s:

$$a{e}^{-t/{\tau }_{1}}+\left(1-a\right){e}^{-\frac{t}{{\tau }_{2}}}+constant,$$

and then follow the same process as the backscatter to calculate the PSD corresponding to the TPF signal (the result from each step is shown in Fig. S13). After determining the PSD in log-scale, we fit the data with the Lorentzian:

$$y\left(f\right)={y}_{0}+\frac{A}{{f}^{2}+{f}_{c}^{2}},$$

Here, ${y}_{0}$ & $A$ are constants, and ${f}_{c}$ corresponds to corner frequency value in log-scale. Note that we have considered the power spectrum in this fitting up to 1 kHz frequency (low-frequency approximation^21,22^). To calculate trap stiffness, we use corner frequency ($({f}_{c})$) following the relation^21–23^:

$${\kappa }_{z}=2\pi \gamma {f}_{c},$$

Here, $\gamma$ is the drag coefficient given by:

$$\gamma =\frac{6\pi \eta r}{1-\frac{9}{16}\left(\frac{r}{h}\right)+\frac{1}{8}{\left(\frac{r}{h}\right)}^{3}-\frac{45}{256}{\left(\frac{r}{h}\right)}^{4}-\frac{1}{16}{\left(\frac{r}{h}\right)}^{5}},$$

where $r=0.5 \mu m$ is the radius of the trapped particle, $\eta =0.85 mPa/s$ is the viscosity of the medium^24^, and $h=50 \mu m$ is the height of trapped particle from the cover glass surface^15^. Using backscatter and TPF signals, we determined the trap stiffness along axial direction at 18.80 mW average power. The calculated corner frequency from backscatter and TPF signals are 154.70 Hz and 287.74 Hz, respectively. The corresponding trap stiffness along the axial direction using backscatter and TPF signals are ~ 7.83 pN/µm and ~ 14.56 pN/µm, respectively. The difference between the trap stiffness values measured from backscatter and TPF signals arises because of photobleaching in TPF leads to more fluctuations (or, higher corner frequency, and hence more trap stiffness, which is erroneous). Since confocal aperture was used for backscatter detection and TPF detection is inherently background free, the fluctuations in the signal must be largely correlated by movement of the trapped bead along axial direction^25^, so the values reported here correspond mainly to axial trap stiffness. The order of values of axial trap stiffness is in agreement with earlier performed experiments with CW lasers^26^. Note that movement of the particle along transverse direction will also contribute to these fluctuations to some extent and the lateral trap stiffness, in principle, can be estimated; this is one key advantage of using confocal detection over optical transmission based experiments (in bright-field imaging mode) which is mostly sensitive to lateral trap stiffness^16^.

Figure 4a shows the TPF and backscatter signals for sequential trapping of two particles. Quite interestingly, when we collect signal at a faster data acquisition rate (400 ns), we see oscillations in backscatter trace when the second particle is being dragged, as shown in Fig. 4b (the inset shows a zoomed-in trace). However, to add to our surprise, no oscillation is observed in the TPF trace. Also, this is not observed in backscatter trace recorded at a slower data acquisition rate (400 µs). When the rise part of the backscatter signal is subtracted and the purely oscillatory residual is plotted in Fig. 4c, from the peak-to-peak position (marked by dotted line in Fig. 4c) it is noticed that the signal has an oscillation period of ~ 3.01 ± 0.36 ms throughout the entire time window. Since the signal must arise from the movement of the second particle (as the first particle is already trapped), it may be inferred that the particle moves with a constant velocity as it is being dragged; this is suggestive of attainment of a terminal velocity inside a medium imposing a viscous drag (similar to terminal velocity experienced by falling rain drops through atmospheric drag). To have a quantitative estimate of this velocity, we consider the total distance (along axial direction) the second particle traverses estimated from the theoretical potential energy surface ($\Delta x$)^16^ and simply divide it by the total time taken (the drag time window) from our experiment ($\Delta t$); this yields the (constant) terminal velocity of the second particle:

$$v=\frac{dx}{dt}\approx \frac{\Delta x}{\Delta t},$$

to be 369.56 ± 31.16 µm⁄s (further discussion is given in Section S3.2 in SI). Therefore, considering the ~3 ms period, the temporal oscillations peak every time the second particle traverses a distance of ~1.1 µm (equivalent to its diameter). Putting all these observations together, we conclude that the oscillations arise due to interference between backscatter amplitudes from the two particles detected by the detector in the far field where the movement of the second particle is controls the interferometric delay; this is schematically depicted in Fig. 4d. Here, the detection of scattering signal from the moving (second) particle is referenced by scattering from the trapped (first) particle, thus both amplitude and phase of the signal is recorded (heterodyne detection). Contrary to backscatter detection, since fluorescence (spontaneous emission) is an incoherent emission, no interference effect is observed in TPF detection (homodyne detection). In other words, these two detection modalities further complement each other as the nature of detection is quite different (heterodyne type vs. homodyne type). Therefore, particle–particle interaction may be explored through backscatter interferometry. However, with the present set-up, it is quite difficult to decipher the specific nature of inter-particle interaction (i.e. Casimir effect, interactions arising from Zeta potential, etc.) which would require improved three-dimensional video microscopy at a much higher frame-rate; alternatively, only the backscatter signal could be tracked with simultaneous wide-field detection and point detection to achieve the required spatiotemporal resolution that has been demonstrated quite recently^27^.

**Figure 4:**
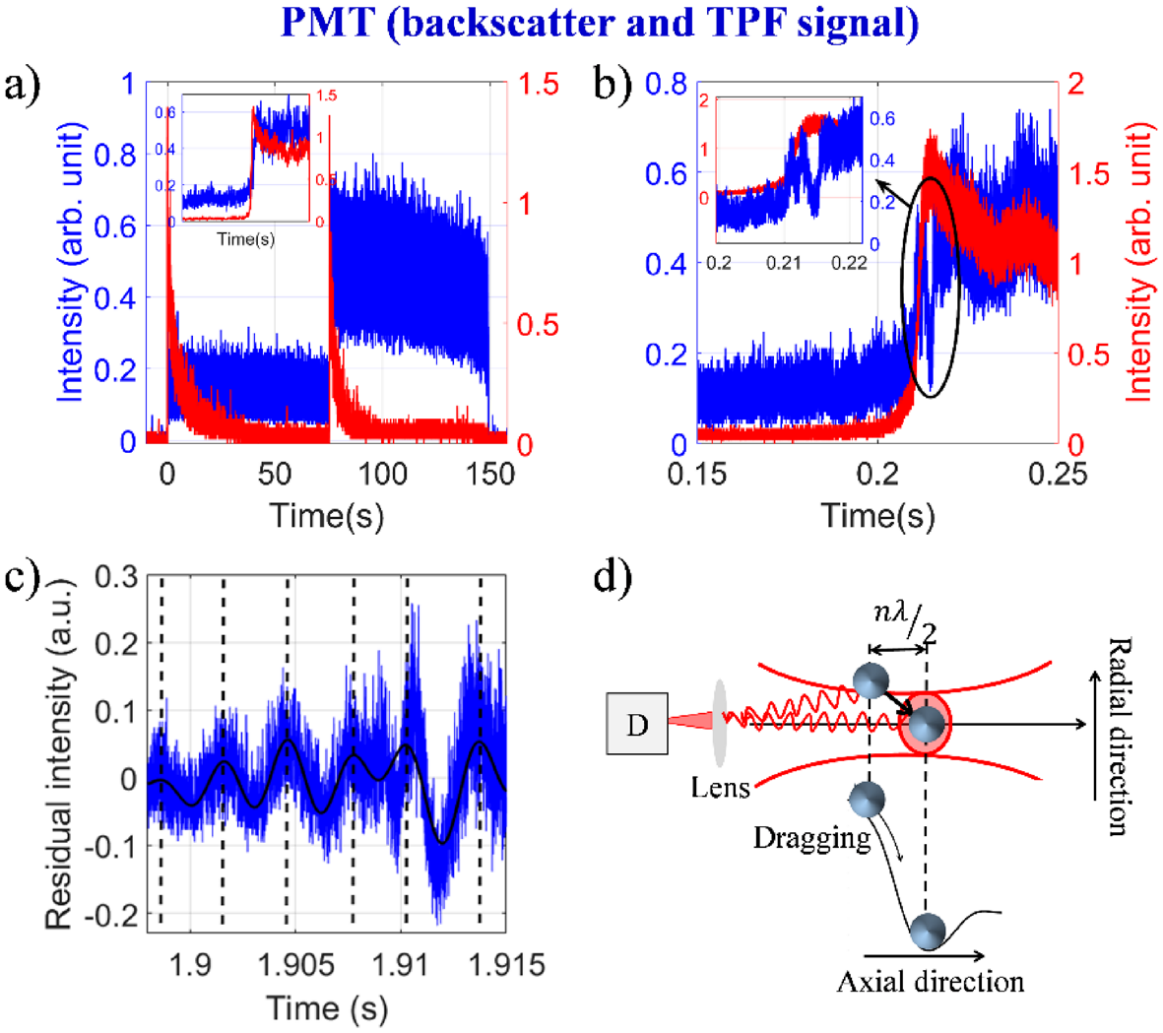
Backscatter and TPF signal (**a**) when two particles confined within the trap and data collection interval is 400 μs; zoomed plot shows the drag of second particle, (**b**) drag of second particle and data collection interval is 400 ns, and (**c**) residual intensity of backscatter signal fitted with Fourier function (black dotted lines represent the peak-to-peak time) at 18.80 mW average power under pulsed excitation. Color: black curve corresponds to Fourier series fitting, red and blue curves correspond to TPF and backscatter signals, respectively. (**d**) Schematic depicting origin of interference pattern during dragging of the second particle when the first particle resides within the nonlinear optical trap.

To summarize, the results presented here demonstrate how complementary detection modalities can capture comprehensive trapping dynamics under femtosecond pulsed excitation. The necessity of spatiotemporal approach of signal analysis is emphasized. Most importantly, the work shows how backscatter detection and TPF detection complement each other in the sense that the shortcomings inherent within one method is overcome by the advantages offered by the other, combined with the fact that the nature of detection is fundamentally different (heterodyne vs. homodyne). These findings are crucial in enriching our understanding of optical trapping dynamics under ultrashort pulsed excitation and promising in having immediate practical applications through controlled optical manipulation.

## Supplementary Information

Supplementary Information.

Supplementary Video 1.

Supplementary Video 2.

Supplementary Video 3.

Supplementary Legends.

## Data Availability

The data that support the findings of this study are available within the article and its Supplementary Material.
