# Supplementary material for: Complementing two-photon fluorescence detection with backscatter detection to decipher multiparticle dynamics inside a nonlinear laser trap
Source: Sci Rep. 2023 Jan 13;13:739. doi: 10.1038/s41598-022-27319-z (PMC9839740; doi:10.1038/s41598-022-27319-z)
Supplement: Supplementary file 1 — Supplementary Information. [file 41598_2022_27319_MOESM1_ESM.pdf]

Complementing two-photon fluorescence detection with backscatter detection to decipher multiparticle dynamics inside a nonlinear laser trap

Anita Devi[a, §], Sumit Yadav[a], and Arijit K. De[b]*

Condensed Phase Dynamics Group, [a]Department of Physical Sciences and [b]Department of Chemical Sciences, Indian Institute of Science Education and Research (IISER) Mohali, Knowledge City, Sector 81, SAS Nagar, Punjab 140306, India.

[§]Present affiliation: Department of Physics, University of Alberta, Edmonton, AB T6G 2R3, Canada.

*akde@iisermohali.ac.in

Contents:

S1. Experiment

S2. Dark-field image analysis using Camera

- **S2.1 Data analysis:**
- **S2.2 TPF detection**
  - **S2.2.1 Single-particle dynamics**
  - **S2.2.2 Multiparticle dynamics**
- **S2.3 Backscatter detection**
  - **S2.3.1 Single-particle dynamics**
    - **S2.3.1.1 Analysis of scatter pattern**
    - **S2.3.1.2 Analysis of total scatter intensity**
  - **S2.3.2 Multiparticle dynamics**
    - **S2.3.2.1 Analysis of scatter pattern**
    - **S2.3.2.2 Analysis of total scatter intensity**

S3. Simultaneous TPF and backscatter detection using PMTs

- **S3.1 Single-particle dynamics**
  - **S3.1.1 Mapping of escape potential**
  - **S3.1.2 Trap stiffness measurement along axial direction**
- **S3.2 Multiparticle dynamics**

S4. References

S1. Experiment

Figure S1 shows a schematic of the experimental set-up for dark-field point-detection mode. For bright-field imaging mode (i.e., wide-field detection mode), light from a white LED is focused on the sample by a condenser in trans-illumination geometry. Figure S2 shows steady-state spectra of the sample used (see Reference 1 for details).

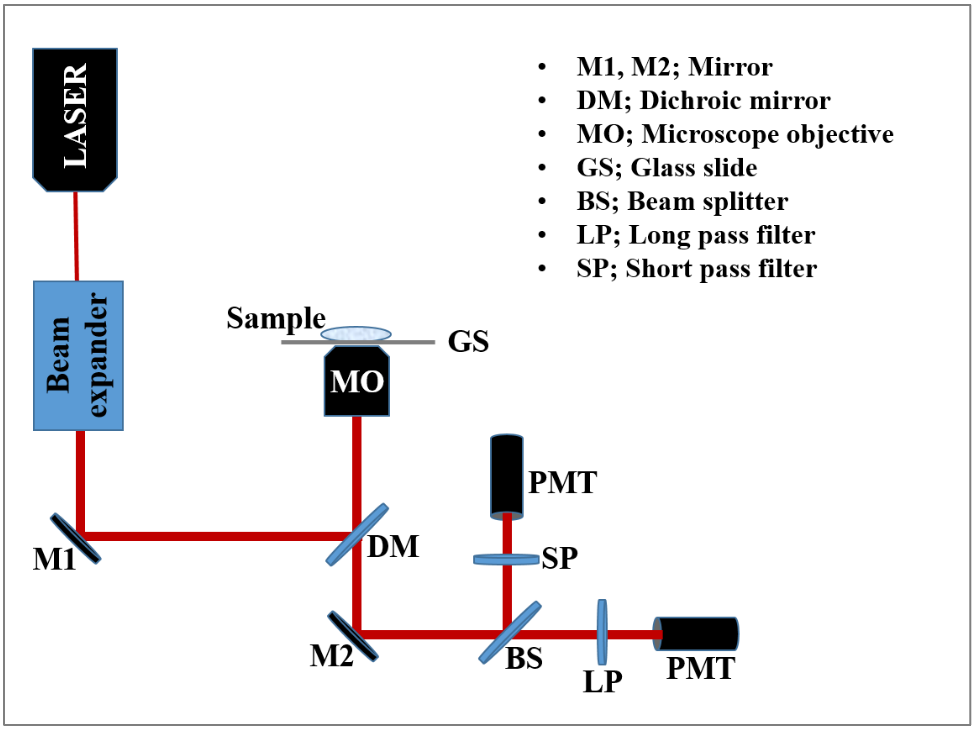

Fig S1. The experimental set-up for for dark-field point-detection mode.

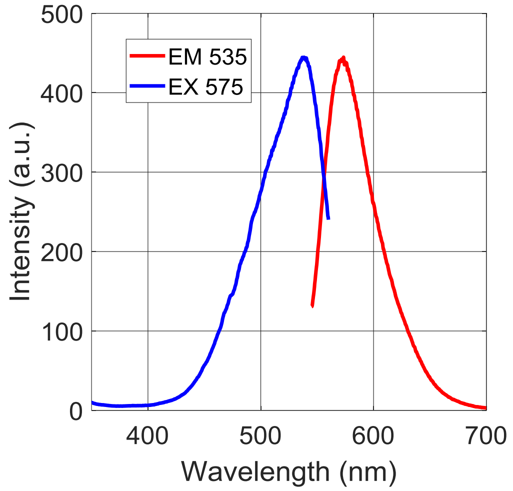

Fig: S2 Fluorescence (emission) spectrum recorded at 535 nm excitation wavelength (red), and fluorescence excitation spectrum recorded at 575 nm emission wavelength (blue). Figure adapted from reference 1.

S2. Dark-field image analysis using Camera

S2.1 Data analysis:

Figures S3a show a two-photon fluorescence image and S3b the corresponding 2D intensity profile of a single-particle confined in a nonlinear optical trap. Figure S3c shows the normalized radial intensity distribution summed along vertical direction over the region of interest (ROI), as shown in figure S3a. Similarly, backscatter data is analyzed; a detailed discussion can be found elsewhere [1]. To analyze the TPF and backscatter signal, we integrate the signal over the center of the rectangular region shown in figure S3c (~15-20 pixel) chosen in Tracker software.

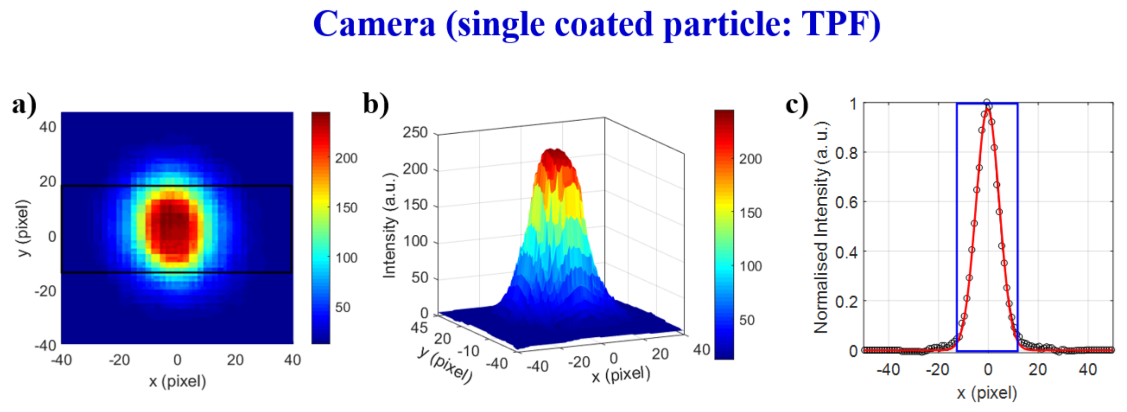

Fig. S3. Plots of TPF a) image, b) 2D intensity profile and c) normalized radial intensity distribution (black) along with Gaussian fit (red) of single-particle inside the optical trap. The area of each pixel is 3.60 x 3.60 μm^2.

S2.2 TPF detection

S2.2.1 Single-particle dynamics

When the particle is dragged to the trap center, there is an immediate rise followed by a decay in the TPF signal over time due to photo-bleaching, as shown in figures S4a-d, and quantitative values are listed in tables S1a-e. The normalized TPF signal decays over time and was fitted to a two-exponential:

$$a \times e^{-\frac{t}{\tau_1}} + b \times e^{-\frac{t}{\tau_2}} \qquad \text{(S1)}$$

using the constraint, $a + b = 1$. The choice of multiexponential is justified by the fact that photobleaching proceeds via sequential steps which involves multiple species. It can be observed that at high average power, the TPF signal shows more fluctuations as compared to low average power because when a laser beam is incident on a polystyrene bead, the local temperature around the particle increases. The temperature distribution follows the Gaussian distribution of the incident laser beam. Due to the inhomogeneous distribution of temperature, the hydrodynamic

force and surface tension varies non-uniformly which results in exerting a net force on the particles that lead to a change in mean square displacement (MSD) of the particle. The local environment's temperature rises with increasing power and the particles experience thermal kicks (from surrounding solvent molecules) to conserve momentum. The Supplementary Video 1 shows one such event in which particle gets photobleached while being trapped.

To further explore the photo-bleaching dynamics, we also study the TPF decay for immobilized particles on the cover-slip. One particle is brought in the focal plane manually and the TPF signal is captured over time and fitted using equation S1. When the particles are immobilized on the surface, they do not experience any thermal kick due to the absence of the solvent. Consequently, no fluctuation is observed, and decays are smooth as shown in figures S4e-f. Tables S2a-b show 10 sets of data fitting parameters at 5.17 mW and 9.40 mW average powers under pulsed excitation and table S3 shows the overall time constant. For a dry sample, the time constants are random as there is always some error associated while bringing the particle in the focal plane. Figure S4g shows the average of all these data sets for both trapped (quantitatively listed in tables S4) and immobilized (quantitatively listed in tables S3) particles and the overall time constant decreases while peak intensity increases with increasing average power which can be seen from figure S4h. For immobilized particles, the data cannot be collected at high average power because it starts melting/ablation [2]; a live event is shown in the Supplementary Video 2.

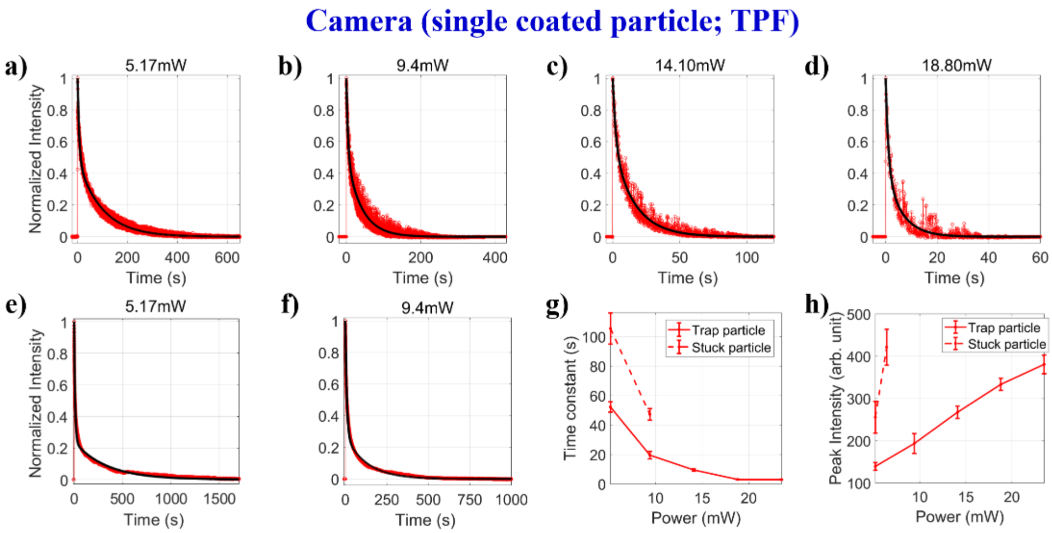

Fig. S4. Plots of a-d) TPF signal over time from a trapped particle, and e-f) plots of TPF signal over time for immobilized particle at different average powers. Plots of g) average TPF photobleaching time constant against average power, and h) peak intensity of TPF signal during trapping against average power for both trapped and immobilized particle. Color: a-f) the red curves correspond to the experimental data, and black curves correspond to the exponential fits.

Peak intensity (a. u.)	a	τ_1 (s)	b	τ_2 (s)	τ_{avg} (s)	R^2
141.59	0.48	97.88	0.52	6.32	50.55	0.98
145.22	0.47	96.8	0.53	5.42	48.85	0.97
139.57	0.49	97.04	0.51	6.52	50.75	0.99
141.56	0.55	82.24	0.45	8.03	48.44	0.99
139.82	0.40	112.2	0.60	11.95	51.88	0.98
144.58	0.48	99.59	0.52	10.24	53.46	0.99
146.87	0.50	92.47	0.50	8.85	50.93	0.99
123.77	0.49	106	0.51	11.78	58.44	0.98
123.10	0.50	105.7	0.50	11.73	58.36	0.99
148.41	0.51	91.68	0.49	8.88	51.02	0.99

Table S1a. Peak intensity and fitting parameters of the TPF signal collected for trapped single-particle at 5.17 mW average power under pulsed excitation.

Peak intensity (a. u.)	a	τ_1 (s)	b	τ_2 (s)	τ_{avg} (s)	R^2
174.08	0.53	39.92	0.47	4.31	23.25	0.97
170.92	0.51	40.07	0.49	4.51	22.73	0.96
182.81	0.53	36.15	0.47	4.95	21.52	0.98
199.13	0.5	34.74	0.5	2.84	18.87	0.93
184.58	0.5	36.96	0.5	4.37	20.77	0.95
173.7	0.51	35.92	0.49	4.13	20.29	0.98
220.63	0.41	37.03	0.59	5.24	18.3	0.96
175.92	0.71	23.17	0.29	2.1	17.05	0.96
235.65	0.51	30	0.49	3.54	16.94	0.96
218.1	0.48	29.57	0.52	3.94	16.14	0.94

Table S1b. Peak intensity and fitting parameters of the TPF signal collected for trapped single-particle at 9.40 mW average power under pulsed excitation.

Peak intensity (a. u.)	a	τ_1 (s)	b	τ_2 (s)	τ_{avg} (s)	R^2
249.16	0.57	16.3	0.43	2.37	10.35	0.97
256.75	0.56	15.59	0.44	2.34	9.80	0.98
261.17	0.54	15.95	0.46	2.47	9.73	0.97
261.58	0.48	16.97	0.52	2.66	9.52	0.95
251.56	0.39	21.87	0.61	3.57	10.71	0.93
271.45	0.51	16.65	0.49	2.5	9.66	0.97
265.47	0.46	18.12	0.54	2.95	9.87	0.96
291.18	0.49	14.73	0.51	2.43	8.45	0.96
288.02	0.51	14.33	0.49	2.66	8.60	0.98
280.11	0.31	19.34	0.69	3.46	8.39	0.96

Table S1c. Peak intensity and fitting parameters of the TPF signal collected for trapped single-particle at 14.10 mW average power under pulsed excitation.

Peak intensity (a. u.)	a	τ_1 (s)	b	τ_2 (s)	τ_{avg} (s)	R^2
322.46	0.66	4.52	0.34	0.5	3.15	0.98
322.46	0.42	6.16	0.58	1.06	3.22	0.93
334.82	0.63	4.79	0.37	0.4	3.18	0.96
321.07	0.67	4.71	0.33	0.56	3.35	0.98
334.52	0.61	4.91	0.39	0.58	3.23	0.97
358.46	0.58	4.71	0.42	0.36	2.9	0.95
322.45	0.74	4.04	0.26	0.18	3.02	0.95
359.22	0.65	4.24	0.35	0.34	2.88	0.98
332	0.61	4.31	0.39	0.52	2.82	0.97
327.07	0.5	5.34	0.5	0.86	3.1	0.97

Table S1d. Peak intensity and fitting parameters of the TPF signal collected for trapped single-particle at 18.80 mW average power under pulsed excitation.

Peak intensity (a. u.)	a	τ_1 (s)	b	τ_2 (s)	τ_{avg} (s)	R^2
358.08	0.66	4.52	0.34	0.5	3.15	0.98
380.32	0.42	6.16	0.58	1.06	3.22	0.93
413.33	0.63	4.79	0.37	0.4	3.18	0.96
361.42	0.67	4.71	0.33	0.56	3.35	0.98
384.63	0.61	4.91	0.39	0.58	3.23	0.97
395.45	0.58	4.71	0.42	0.36	2.9	0.95
406.98	0.74	4.04	0.26	0.18	3.02	0.95
379.21	0.65	4.24	0.35	0.34	2.88	0.98
341.48	0.61	4.31	0.39	0.52	2.82	0.97
387.05	0.5	5.34	0.5	0.86	3.1	0.97

Table S1e. Peak intensity and fitting parameters of the TPF signal collected for trapped single-particle at 23.50 mW average power under pulsed excitation.

Peak intensity (a. u.)	a	τ_1 (s)	b	τ_2 (s)	τ_{avg} (s)	R^2
209.76	0.30	324.6	0.71	16.37	107.30	0.99
287.30	0.24	332.2	0.76	9.95	87.42	0.97
298.40	0.24	374.6	0.76	10.25	95.87	0.97
256.27	0.31	307.6	0.70	15.24	104.30	0.98
277.80	0.28	337.4	0.73	14.76	103.49	0.96
232.10	0.26	411.7	0.74	15.35	118.04	0.96
233.30	0.30	326.5	0.70	10.63	104.13	0.97
291.18	0.26	370	0.74	10.57	105.46	0.96
190.60	0.44	261.2	0.56	19.32	125.94	0.99
280.04	0.29	326.2	0.71	14.98	104.18	0.98

Table S2a. Peak intensity and fitting parameters for the TPF signal collected for immobilized single-particle for 5.17 mW average power.

Peak intensity (a.u.)	a	τ_1 (s)	b	τ_2 (s)	τ_{avg} (s)	R^2
436.04	0.27	128.8	0.73	8.55	41.33	0.99
375.91	0.27	167.6	0.73	8.83	52.25	0.98
364.17	0.31	152.2	0.69	8.34	52.73	0.99
405.14	0.21	199	0.78	8.55	49.52	0.94
465.25	0.25	161.1	0.74	6.09	45.52	0.98
449.33	0.26	159.6	0.74	8.05	47.21	0.98
392.54	0.25	154.5	0.75	9.27	45.68	0.98
433.20	0.30	153.4	0.70	6.68	50.48	0.98
498.94	0.33	116.9	0.67	4.45	42.01	0.98
394.69	0.28	140.9	0.72	8.33	45.52	0.99

Table S2b. Peak intensity and fitting parameters for the TPF signal collected for immobilized single-particle for 9.40 mW average power.

Power (mW)	Peak Intensity (a. u.)	τ_{avg} (s)
5.17	255.67 ± 37.37	105.61 ± 10.58
9.40	421.52 ± 42.46	47.22 ± 3.97

Table S3. Average peak intensity and time constants for 10 sets of data (tables S2a-S2b) for immobilized single-particle at different average power.

Power (mW)	Peak intensity (a.u.)	τ_{avg}
5.17	139.45 ± 8.93	52.30 ± 3.52
9.4	193.55 ± 23.41	19.6 ± 2.50
14.1	267.65 ± 14.70	9.51 ± 0.79
18.8	333.45 ± 14.34	3.09 ± 0.17
23.5	380.79 ± 22.24	2.98 ± 0.24

Table S4. Average peak intensity and time constants for 10 sets of data (tables S1a-S1e) for trapped single-particle at different average power under pulsed excitation.

S2.2.2 Multiparticle dynamics

The multiparticle trapping dynamics using TPF signal from camera is already discussed in the main text.

S2.3 Backscatter detection

S2.3.1 Single-particle dynamics

S2.3.1.1 Analysis of scatter pattern

To determine particle motion with respect to polarization direction, we further analyze the scattering pattern of single-particle (a detailed discussion on scattering pattern as shown in figure S5a can be found elsewhere [1]). It is observed that the intensity of the signal from each of these four lobes varies differently with time (figure S5b-e) compared with the overall intensity, i.e. integrated over all the four lobes (figure S5f). At different time windows, the intensity of any two lobes may vary in a correlated or anti-correlated or un-correlated fashion (figure S5g-i). This is because when the particle is trapped, it may have motion along all possible radial directions inside the optical trap (figure S5j). For example, figure S5i shows the intensity of lobe 3 and lobe 4 are correlated over a fixed time window, suggesting that the particle most likely moves along path M1 (figure S5k). Similarly, when the particle moves along M2, there is a correlation between intensities of lobe1 and lobe4 while the other two (lobe2 and lobe3) may or may not have any correlation (figure S5l). From the intensity of lobes, we can determine the direction of particle motion at any instant of time (the more intense lobe indicates the direction of motion of particle among the infinite number of motions, as shown in figure S5j).

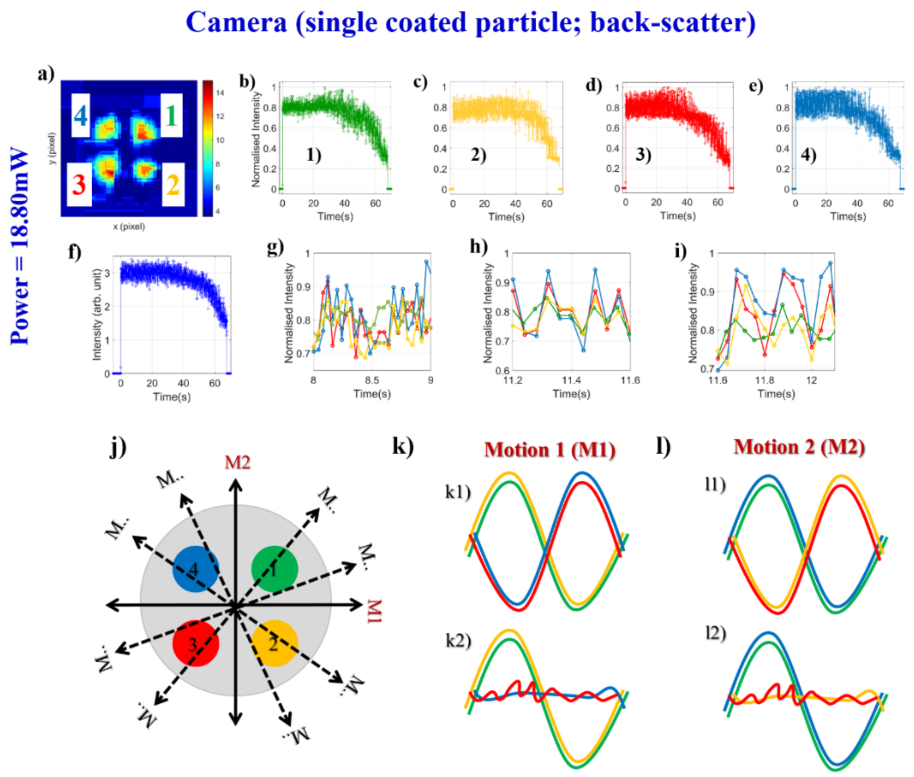

Fig. S5 a) Contour plot of backscatter signal from a trapped particle; b-e) time traces of intensity of individual lobes of backscatter signal (shown in figure a); f) time trace of the integrated intensity of all four lobes (shown in figure a); g-i) plots of zoom-in time trace of individual lobes; j) a proposed model for the trapped particle's lateral trajectories giving rise to particular intensity fluctuations; k-l) a motion along path M1 leads to correlated fluctuations of intensities lobe 3 and lobe 4 while that of lobe 1 and lobe 2 may be correlated (k1) or have no correlation (k2) and vice-versa; similarly, motion along path M2 can be predicted. Color: blue/green/yellow/red/light blue corresponds to the backscatter of all four-lobes/ lobe1/lobe2/lobe3/lobe4, respectively.

S2.3.1.2 Analysis of total scatter intensity

Single-particle backscatter signal integrated over all the four-lobe at different average power under both CW and pulsed excitation are shown in figure S6. It is found that the confinement time (total time for which the particle stays inside the trap) changes with average power. The backscatter shows a sharp rise followed by a plateau and eventually decays gradually under pulsed excitation, whereas, under CW excitation, backscatter persists for the entire observation time window. The plateau's time range decreases with an increase in average power under pulsed excitation, as shown in figure S6B. The noticeable point here is that at 5.17 mW average power, the particle's confinement time is more than 2000 sec (figure S6A). However, under similar conditions, the TPF signal lasts only for 600 sec due to photobleaching (figure S4). Similarly, for other average powers, it is difficult to get information on confinement time from the TPF signal. In contrast, the backscatter signal shows a sudden drop in the signal as the particle leaves the

trap. It is also observed that fluctuations in the signal increase with time along the radial as well as axial directions owing to more anharmonic nature of the trap.

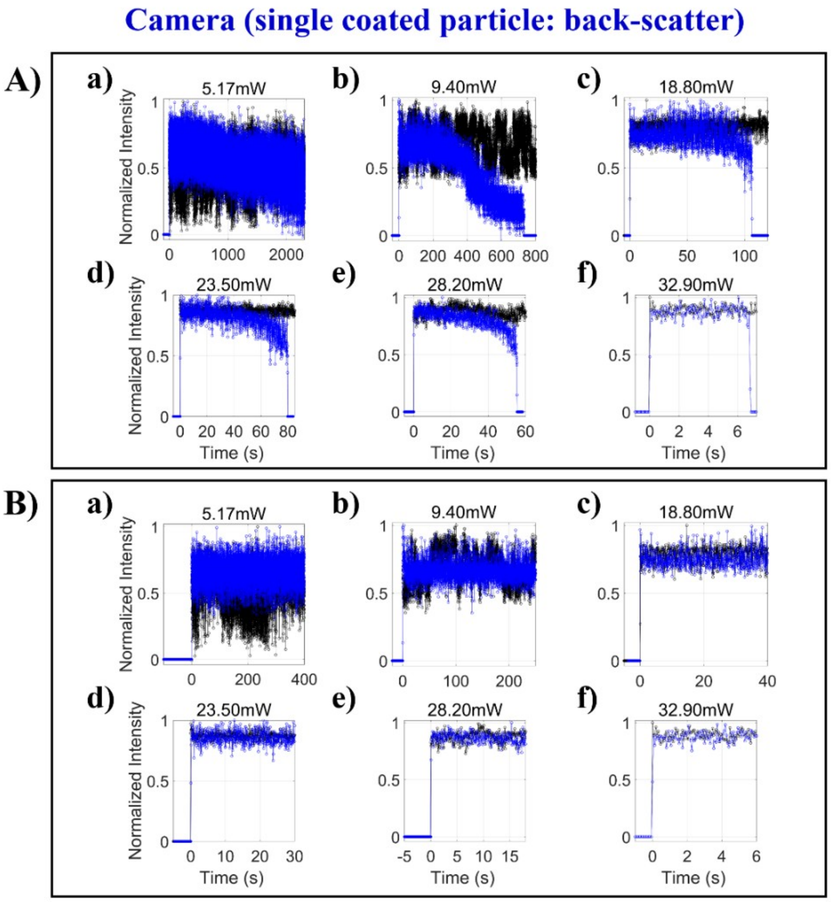

Fig. S6. Plots of backscatter signal of single-particle shown for A) overall time window of trapping event, and B) zoomed-in plateau region under both CW and pulsed excitation in which a) at 5.17 mW, b) at 9.40 mW, c) at 18.80 mW, d) at 23.50 mW, e) at 28.20 mW, f) at 32.90 mW average power, respectively. Color: black curves correspond to CW excitations, and blue curves correspond to pulsed excitation.

S2.3.2 Multiparticle dynamics

S2.3.2.1 Analysis of scatter pattern

The dynamics of two particles inside the trap involve a collision between the particles. Depending on the momentum carried by the incoming second particle and the inter-particle interaction, the particles can be arranged in many possible configurations inside the optical trap, as shown in figure 2 (main text). The first row in figure 2 (main text) represents the time-series backscatter images when two particles are confined within the optical trap. The second row is a radial (top) view of the particle inside the optical trap with respect to reference particle (P1). The third row shows a proposed 3D representation of two particles inside the trap. The fourth row

shows the different configurations of the two confined particles inside the asymmetric potential well. The first column represents the backscatter pattern in which one particle resides in the trap after that second particle is superimposing on the first, making all the four lobes equally intense along with a first-order diffraction ring. The projection of overlapping region of the two particles determines the intensity of lobes. This observation is confirmed by the proposed scheme in the second, third, and fourth row.

Further to understand the dynamics, we analyze the backscatter signal, as shown in figure S7a (image), and figure S7b-e shows the individual time trace signal of the four lobes marked in the figure S7a, and it can be observed that all four lobes have different decay. Please note that decay signals of lobes 1 and 3 look similar, showing oscillatory nature, and that of lobes 2 and 4 look similar, which reveals some degree of correlation between the lobes. Figure S7f shows the total backscatter (integrated over all four lobes); the decay observed in this case is similar to that of lobes 2 and 4. This is because lobes 1 and 3 are anti-correlated, resulting in the mutual cancellation of their contributions to the total backscatter. This correlation behavior of the two sets of the lobes is further illustrated by zoomed-in time trace shown in figures S7g-j, where figure S7g shows the time window for lobes 1 and 3, which are anti-correlated, while figure S7h shows that lobes 2 and 4 are not correlated over the same time window. Subsequently, lobes 1 and 3 as well as 2 and 4 become anti-correlated, as shown in figures S7i-j. Thus, it is observed that decay in lobes 1 and 3 are anti-correlated, while lobes 2 and 4 have a random correlation in different time windows of trapping time. With time, the pairing of lobes which are correlated, is shuffled or there may not be any correlation between any pair at all.

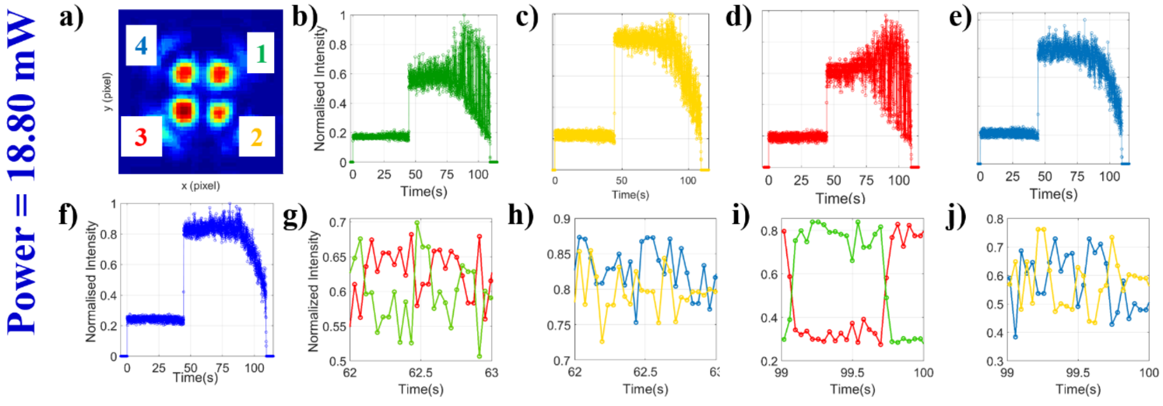

Fig. S7. a) Image of backscatter from an optically trapped two particles under pulsed excitation. b-e) Time traces of intensity of individual lobe of the scatter pattern (shown in figure a) and f) time trace of the integrated intensity of all four lobes (shown in figure a). Time traces for g) time window where lobes 1&3 are anti-correlated, h) same time window of i where lobes 2&4 have no correlation; i) another time window where lobes1&3 are anti-correlated, and j) lobes 2&4 are anti-correlated but both have no correlation. Color: blue/green/yellow/red/light blue corresponds to the backscatter all four lobes/ lobe1/lobe2/lobe3/lobe4 respectively.

S2.3.2.2 Analysis of total scatter intensity

Figure S8a-c shows backscatter signal (integrated over four lobes) for two particles confined in the trap where an immediate rise in the signal indicates the second particle's trapping. It can be observed that the confinement time for second particles decreases with increasing average power. This is strongly dependent upon the height of escape potential, which decreases with average power within this limit, consequently, the residence time of the second particle inside the trap decreases. Figure S8d shows a proposed model for dragging of the second particle inside the potential well where the first particle is already trapped. For confining the second particle inside the trap, the first particle has to shift its position (either forward or backward, depending upon the direction from which the second particle is dragged). The two particles may adapt to many possible configurations, as shown in figure 2 (main text). Unlikely, both particles can go out from the trap, or the first particle can go out from the trap and second particle may stay there. However, at a similar average power, the second particle's arrival time within the focal volume also matters. For example, if the second particle is dragged when the first particle is about to eject from the trap, at that time, the first particle needs very small momentum to escape from the trap. Consequently, when the second particle is dragged within the focal volume as the first particle leaves the trap, as shown in figure S8e. If the second particle dragged earlier, then the particles' confinement time depends upon the escape potential, (which, in turn, depends upon the average power). If the momentum carried by the second particle is too high, both particles may get ejected from the trap, as shown in figure S8f There might be a possibility that instead of two particles, only one will cross the barrier and the other one is retained, as shown in figure S8g.

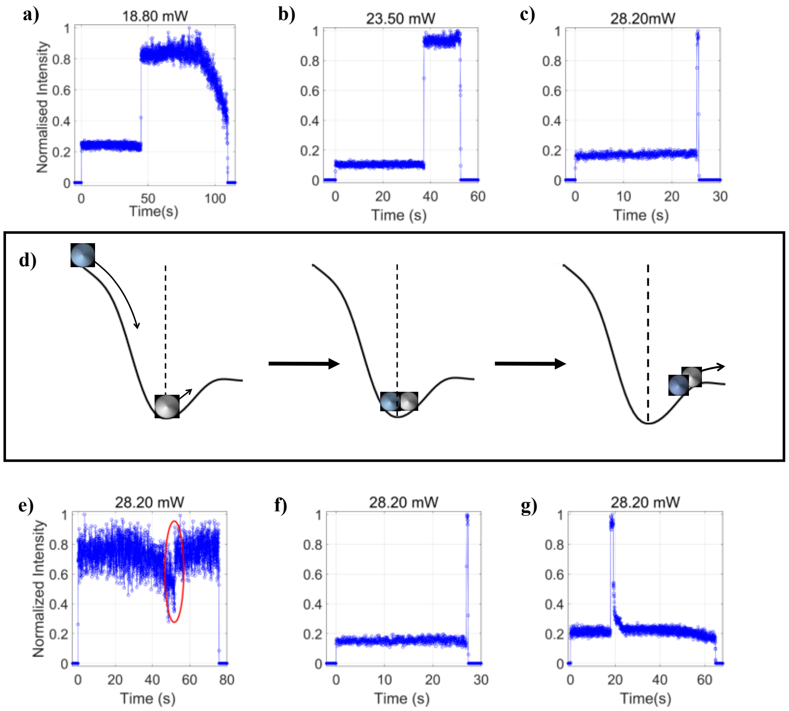

Fig. S8. The backscatter (integrated over all four lobes) during trapping of two particles under pulsed excitation; a) at 18.80 mW, b) at 23.50 mW, and c) at 28.20 mW average power. d) A proposed model when the second particle is drag inside the focal volume while one particle is already confined within the optical trap. e) The second particle dragged when one particle is about to leave so due to collision one is retained but other leaves the trap, f) the second particle is dragged when particle is settled at equilibrium position and start leaving the trap but after collision both went out, and g) the second particle is dragged after first but due to small escape potential, two particles cannot stay together so one is ejected at 28.20 mW average power.

Figure S9 shows the backscatter signal (integrated over four lobes) for trapping of multiple particles inside the nonlinear laser trap. At any instance, more than two particles cannot stay inside the trap. The circles in figure S9a shows a kink in the backscatter which can be explained by the corresponding proposed model shown in figure S9b. The proposed model suggests that the first rise in the signal indicates the trapping of the first particle, and the second rise indicates the dragging of the second particle. The third incoming particle collides with the two existing particles and one of them is ejected. Eventually, both particles go out from the trap, which results in an immediate drop in the scattering signal. In general, the incoming particle collides with existing particles that will result in the ejection of one/two/all particle(s) depending upon the collision between particles which again depends on the average power of trapping beam. The higher the average power lesser will be the probability of finding particle(s) in the trap since escape potential decreases significantly. For example, another possible case can be seen in figure S9c, which shows the backscatter (integrated over all four lobes) for multiple particles drag inside the trap at 28.20 mW average power under pulsed excitation. This scattering signal can be well explained by the proposed model shown in figure S9d. The model shows that initially one

particle is dragged which results in a rise in the signal, after some time, another particle is dragged, but due to collision one of the particles leaves the trap. When another particle is dragged both the particles stay inside the trap for a while before being ejected together.

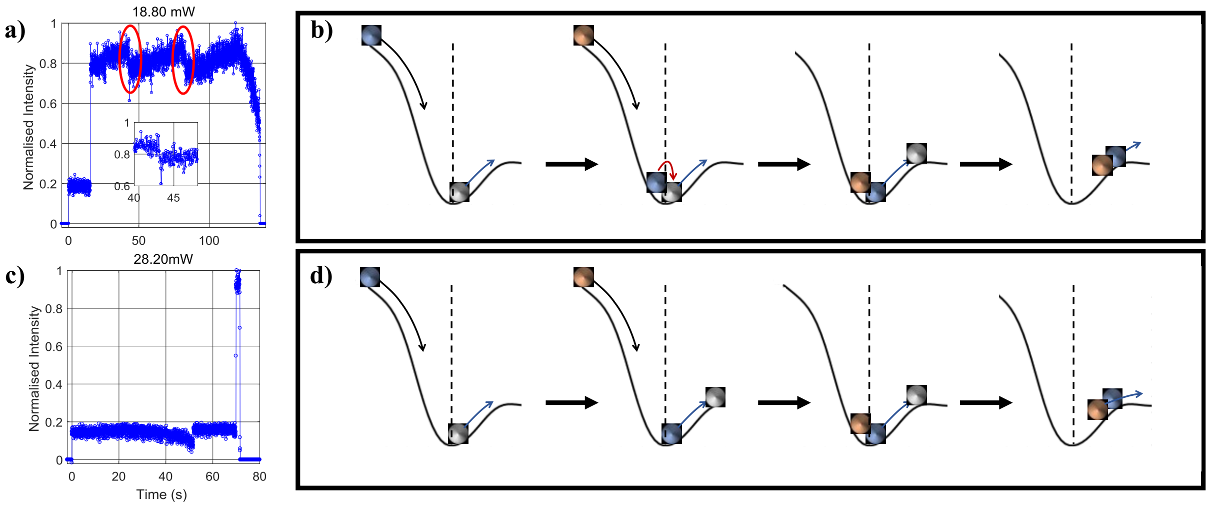

Fig. S9. Dynamics of trapping of multiple particles under pulsed excitation: a) backscatter (integrated over all four lobes) at 18.80 mW average power, b) proposed model corresponding to a; c) backscatter (integrated for all four lobes) at 28.20 mW average power; d) proposed model corresponding to c.

S3. Simultaneous TPF and backscatter detection using PMTs

S3.1 Single-particle dynamics

Figure S10a shows the TPF signal for a single trapped particle against real-time. The normalized TPF signal was fitted using three exponential which can be expressed as:

$$a \times e^{-\frac{t}{\tau_1}} + b \times e^{-\frac{t}{\tau_2}} + c \times e^{-\frac{t}{\tau_3}} + d \qquad \text{(S2)}$$

by using the constraints, $a + b + c + d = 1$.

Figure S10b shows the value corresponded to the maximum rise in the TPF signal against average power when the particle dragged within the optical trap. It can be observed that TPF maxima increase with an increase in average power because the maximum intensity of the TPF signal is proportional to average power. While a slope of 2 of the linear fit to the log-log plot is expected for a two-photon process (compared with a slope of 1 for a one-photon process), the data shows a slope in-between 1 and 2 suggestive of increased photobleaching with an increase in power. Figure S10c shows the average time constant of the TPF signal for three exponentials against average power. The overall time constant decreases with an increase in average power, quantitatively listed in table S5-S6. Consequently, the process of photo-bleaching in TPF occurs faster at high average power which results in decreasing the overall time constant and increasing

the peak intensity. For fixed average power, the overall time constant for the TPF signal increases while intensity maxima decrease with the increase in the number of raw data points for moving-averaging, quantitatively listed in tables S5-S6. Thus, an increasing number of data points for the moving-averaging results in losing the system's original information which can be seen from figures S10b-c.

Figure S10d-i shows single-particle behavior confined by the asymmetric potential at different average power under both CW and pulsed excitation. In the TPF signal, sudden rise followed by the decay contains information on drag and adjustment times of the trapped particle. After a certain time when the particle gets photobleached, a residual fluorescence signal is present. Although the residual fluorescence signal is very less and it is difficult to get useful information from raw data, it stays until the particle is confined within the optical trap. The information on residual signal can be gathered by moving-averaging of raw data and observed that residual signal increases with average power, quantitatively listed in table S6.

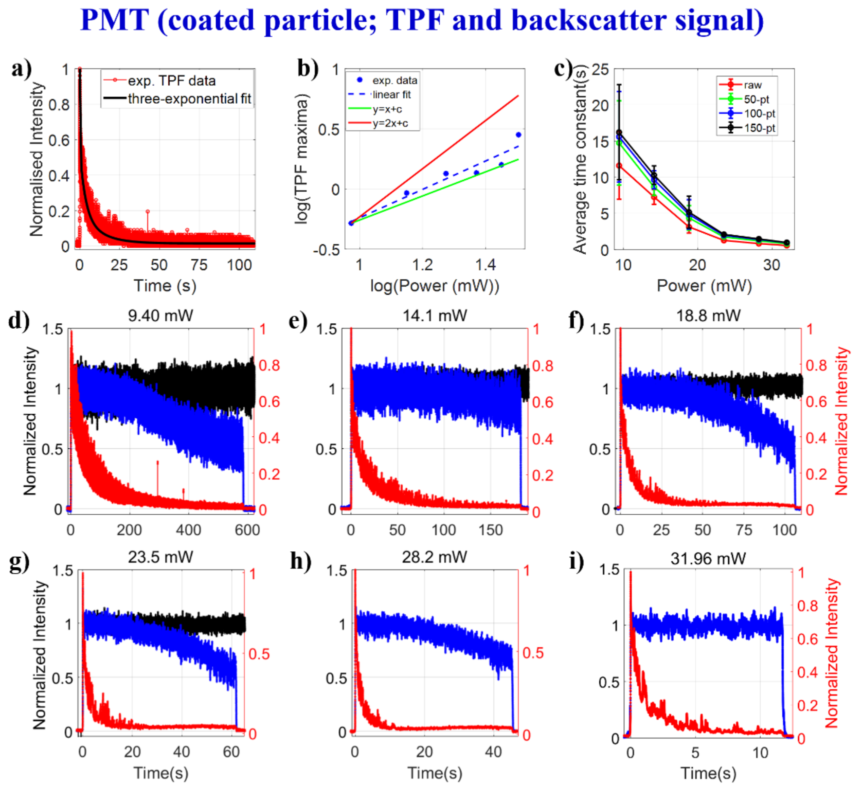

Fig. S10. Plots of a) TPF signal over time for single-particle under pulsed excitation, b) maximum value of TPF signal when the particle is dragged to the optical trap against average power along with linear fit (blue, dashed line) where we also show lines with slopes 2 (solid, red) and 1 (solid, green), and c) average TPF photobleaching time constant against average power for trapped particle. The plots of backscatter and TPF signal at 50-pt moving averaging against real-time under both CW and pulsed excitation for single-particle at d) 9.40 mW, e) 14.10 mW, f) 18.80 mW, g) 23.50 mW, h) 28.20 mW, and i) 31.96 mW average power. Color: black corresponds to backscatter under CW excitation, blue and red correspond to backscatter, and TPF signal under pulsed excitation.

Power (mW)	Three exponentials						
	a	τ_1 (s)	b	τ_2 (s)	c	τ_3 (s)	Residual TPF Signal
9.40	0.23 ± 0.09	41.09 ± 9.91	0.35 ± 0.06	5.62 ± 2.13	0.41 ± 0.04	0.0256 ± 0.0154	0.0084 ± 0.0122
14.10	0.28 ± 0.05	22.28 ± 4.68	0.28 ± 0.04	3.94 ± 1.39	0.43 ± 0.02	0.0204 ± 0.0060	0.0114 ± 0.0011
18.80	0.26 ± 0.06	9.72 ± 2.26	0.31 ± 0.06	1.89 ± 0.61	0.42 ± 0.04	0.0153 ± 0.0066	0.0139 ± 0.0027
23.50	0.26 ± 0.09	4.20 ± 0.76	0.27 ± 0.08	0.78 ± 0.44	0.46 ± 0.03	0.0093 ± 0.0028	0.0162 ± 0.0017
28.20	0.22 ± 0.07	2.89 ± 0.66	0.29 ± 0.07	0.68 ± 0.27	0.47 ± 0.04	0.0088 ± 0.0023	0.0161 ± 0.0019
31.96	0.28 ± 0.07	1.85 ± 0.69	0.24 ± 0.06	0.30 ± 0.17	0.47 ± 0.06	0.0076 ± 0.0021	0.0160 ± 0.0193

Table S5. Fitting parameters for TPF signal for trapped single-particle at different average power for raw data using three exponential fit.

Power (mW)	Three exponentials (τ_{avg} (s))			
	Raw data	50-pt moving-averaging	100-pt moving-averaging	150-pt moving-averaging
9.40	11.61 ± 4.66	14.75 ± 5.83	15.60 ± 6.26	16.22 ± 6.59
14.10	7.27 ± 1.02	8.66 ± 1.18	9.64 ± 1.25	10.30 ± 1.27
18.80	3.13 ± 0.86	4.38 ± 1.69	4.86 ± 2.04	5.17 ± 2.21
23.50	1.27 ± 0.05	1.74 ± 0.06	1.96 ± 0.08	2.09 ± 0.08
28.20	0.83 ± 0.04	1.20 ± 0.13	1.38 ± 0.16	1.47 ± 0.13
31.96	0.56 ± 0.13	0.81 ± 0.19	0.95 ± 0.21	0.97 ± 0.20

Table S6. The average time constant of TPF signal for trapped single-particle at different average power for raw data, 50-pt, 100-pt, and 150-pt moving-averaging of raw data using three exponential fittings.

S3.1.1 Mapping of escape potential

As we discussed above, with increasing average power, confinement time for particle decreases (figure S11a), and eventually, trap becomes unbound at high average power [3]; therefore, the particle cannot be trapped. The confinement time is strongly dependent on the asymmetric nature of the optical trap which increases with the average power of the trapping beam. In order to map the escape potential, we assume that the reciprocal of the confinement time (t_{con}), which is the rate constant (k_{con}) at which it escapes the one-dimensional barrier (along axial direction), follows Arrhenius-like behavior ($k_{con} \equiv \frac{1}{t_{con}} \propto e^{-U_{esc}/k_B T}$). Figure S11b shows the escape potential variation with power from confinement time, which results in a qualitative agreement in the high-power fall-off region with earlier work [3].

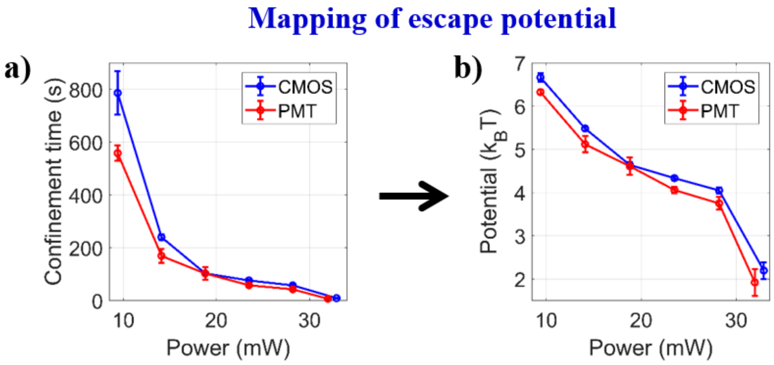

Fig. S11. Plots of a) confinement time against average power and b) escape potential against average power.

S3.1.2 Trap stiffness measurement along axial direction

Determining the trap stiffness along the axial direction is crucial, therefore, we propose that by using TPF and backscatter signals, trap stiffness along the axial direction can be measured as both the signals are detected using the point detection method. Since backscatter signal is collected using confocal aperture the signal's fluctuation corresponds to the particle motion along the axial direction. The same is also true for TPF detection because of its built-in confocality. Therefore, we considered both backscatter and TPF signals when the particle is trapped, as shown in figures S12 and S13.

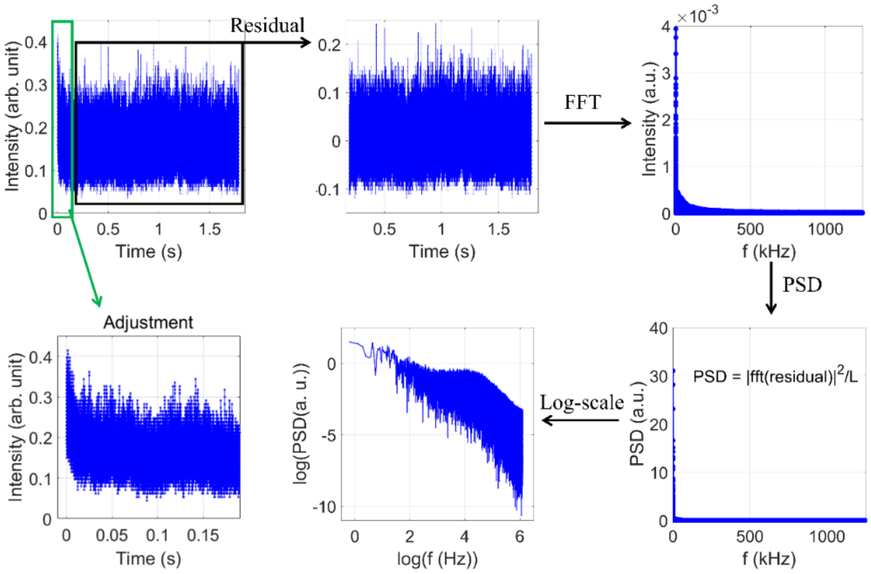

Fig. S12 Plots showing step-by-step analysis of trap stiffness along axial direction using backscatter signal from a single trapped particle at 400 ns time interval.

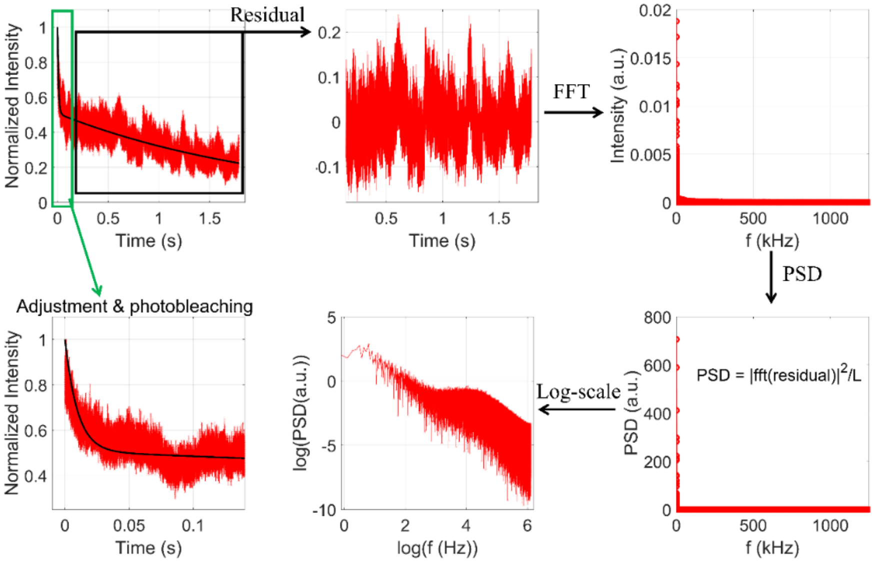

Fig. S13 Plots showing step-by-step analysis of trap stiffness along axial direction using two-photon fluorescence signal from a single trapped particle at 400 ns time interval.

S3.2 Multiparticle dynamics

As discussed earlier, Figure S8d shows a proposed model for the second particle's dragging, where one particle is already confined in the trap. For confining the second particle inside the trap, the residing particle has to shift its position inside the optical trap. Afterward, it can adjust

itself in many possible configurations, as shown in figure 2 (main text). As shown in figure S14, we see similar dynamics as in figure 2 (main text) but with much improved time resolution.

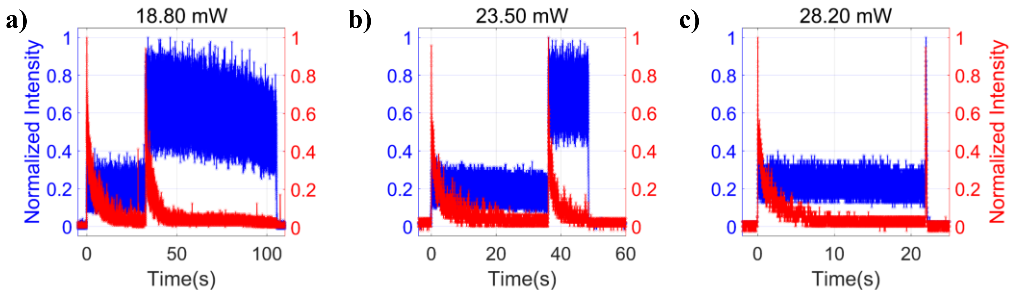

Fig. S14. The plot of simultaneous detection of backscatter and TPF signal during trapping of two particles under pulsed excitation h) at 18.80 mW, i) at 23.50 mW, and j) at 28.20 mW average powers. Color: red and blue curves correspond to TPF and backscatter signals, respectively.

We also analyze the backscatter signal recorded at 400 ns time interval. Figure S15a shows the TPF and backscatter signals when the second particle is being trapped. The noticeable point here is that when data is collected at 400 μs time intervals, the backscatter signal's rise is smooth when the second particle is dragged, however, at 400 ns time interval, we see the interference pattern. We further analyze this signal to estimate the velocity of the second particle while being dragged. To find the velocity (v) of the second particle during drag, we estimate the total time (Δt) taken from Sigmoidal fit to the rise in TPF signal, as shown in figure S15b. To estimate the total distance (Δx) traversed during this time, we use the Sigmoidal fit to numerical results (as shown in figure S15c) using exact Mie theory and including optical as well as thermal nonlinearities [3]. From the residual oscillating backscatter signal (after subtracting the Sigmoidal fit, as shown in figure S15d), we see that the oscillations are temporally equispaced, as shown in figure S15e. Therefore, the particle is dragged with a constant velocity (i.e. the terminal velocity inside a medium) and table S7 contains the calculated velocity for different average powers. The Fourier transform of this oscillatory signal shows a band of frequencies between 200 Hz and 800 Hz, as shown in the inset of figure S15f.

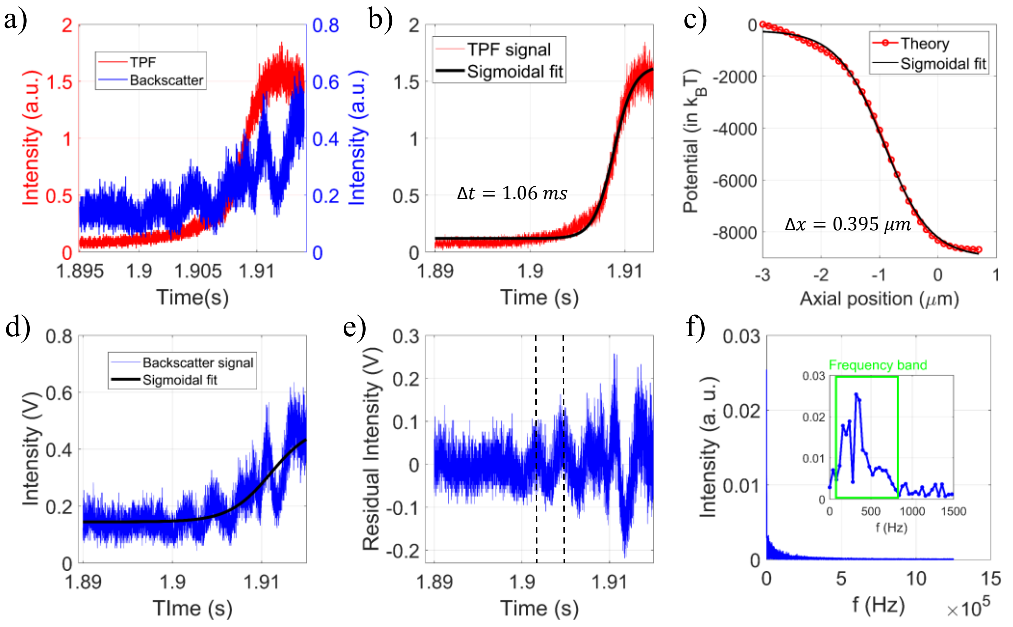

Fig. S15. Plots for a) simultaneous signal of both backscatter and TPF when the second particle is dragged at 18.80 mW average power under pulsed excitation. Data is collected at 400 ns time interval. Plots of b) TPF signal fitted with sigmoidal and c) numerically simulated potential against axial position using exact Mie theory along with Sigmoidal fit. Plots of d) backscatter signal fitted with sigmoidal, e) residual signal of backscatter signal, and f) Fourier transformation of the residual signal.

Power (mW)	$\Delta t\ (ms)$	$\Delta x\ (nm)$	Velocity $(\mu m/s)$
14.10	1.44±0.05	398.56	277.40±10.14
18.80	1.08±0.09	395.14	369.56±31.16
23.50	0.86±0.15	392.81	467.66±79.59

Table S7. Lists the average power and the corresponding backscatter signal dragging time for the second particle, the spatial coordinate of dragging time, and the second particle velocity while dragging within the nonlinear optical trap under pulsed excitation.

S4. References

1. A. Devi, and A. K. De, "A table-top compact multimodal nonlinear laser tweezer," Opt. Commun. **482,** 126440 (2020).

2. V. S. Vasilev, and R. V. Skidanov, "Microexplosions polysterene microparticles on substrate covered by aluminum," Information Technology and Nanotechnology 2017.

3. A. Devi, S. Yadav, and A. K. De, "Dynamics of a dielectric microsphere inside a nonlinear laser trap," Appl. Phys. Lett. **117(16)**, 161102 (2020).
