# Supplementary material for: Complementing two-photon fluorescence detection with backscatter detection to decipher multiparticle dynamics inside a nonlinear laser trap
Source: Sci Rep. 2023 Jan 13;13:739. doi: 10.1038/s41598-022-27319-z (PMC9839740; doi:10.1038/s41598-022-27319-z)
Supplement: Supplementary file 5 — Supplementary Legends. [file 41598_2022_27319_MOESM5_ESM.docx]

Video 1: Live footage of decay of two-photon fluorescence signal due to photobleaching during optical trapping of a coated particle at 18.80 mW average power under pulsed excitation.

Video 2: Live footage of melting of an immobilized particle stuck on cover slip at 18.80 mW average power under pulsed excitation. We have made the video 2 times slower than the original video.

Video 3: Live footage of back scatter signal pattern during optical trapping of coated particles at 18.80 mW average power under pulsed excitation; initially one particle was trapped followed by trapping of a second one. We have made the video 8 times faster than the original video.
